# Burn injury characteristics, referral pattern, treatment (costs), and outcome in burn patients admitted to a hospital with or without a specialized Burn Centre (BURN-Pro)

**DOI:** 10.1007/s00068-023-02233-9

**Published:** 2023-02-03

**Authors:** Daan T. Van Yperen, Esther M. M. Van Lieshout, Margriet E. Van Baar, Suzanne Polinder, Michael H. J. Verhofstad, Cornelis H. Van der Vlies, BURN-Pro Study Group, Anne Y. M. V. P. Cardon, Piet A. R. De Rijcke, Marc Guijt, Taco M. A. L. Klem, Koen W. W. Lansink, Bas J. Punt, Akkie N. Ringburg, Maarten Staarink, Alexander H. Van der Veen, Percy V. Van Eerten, Floortje C. Van Eijck, Paul A. Vegt, Dagmar I. Vos, Marco Waleboer

**Affiliations:** 1 Trauma Research Unit, Department of Surgery, Erasmus MC, University Medical Centre Rotterdam, P.O. Box 2040, 3000 CA Rotterdam, The Netherlands; 2 Burn Centre, Maasstad Hospital, Rotterdam, The Netherlands; 3 Association of Dutch Burn Centres, Burn Centre, Maasstad Hospital, Rotterdam, The Netherlands; 4 Department of Public Health, Erasmus MC, University Medical Centre Rotterdam, Rotterdam, The Netherlands

**Keywords:** Burn care, Treatment, Outcomes, Non-burn centre hospital

## Abstract

**Purpose:**

Data on the epidemiology, treatment, and outcome of burn patients treated at non-burn centre hospitals are not available. The primary aim was to compare the burn characteristics of patients admitted to a hospital with or without a specialized burn centre.

**Methods:**

This multicentre, prospective, cohort study enrolled patients with burns admitted to a hospital without a burn centre and patients with < 10% total body surface area (TBSA) burned admitted to the burn centre. Primary outcome measure was the burn-related injury characteristics. Secondary outcome measures were adherence to the Emergency Management of Severe Burns (EMSB) referral criteria, treatment (costs), quality of life, and scar quality.

**Results:**

During the 2-year study period, 48 patients were admitted to a non-burn centre and 148 patients to the burn centre. In the non-burn centre group, age [44 (P_25_–P_75_ 26–61) versus 30 (P_25_–P_75_ 8–52) years; *P* = 0.007] and Injury Severity Score [2 (P_25_–P_75_ 1–4) versus 1 (P_25_–P_75_ 1–1); *P* < 0.001] were higher. In the burn centre group, the TBSA burned was significantly higher [4% (P_25_–P_75_ 2–6) versus 2% (P_25_–P_75_ 1–4); *P* = 0.001], and more surgical procedures were performed (in 54 versus 7 patients; *P* = 0.004). At 12 months, > 85% of the non-burn centre group and > 75% of the burn centre group reported no problems in quality of life. Scar quality score was < 1.5 in both groups, with significantly poorer scores in the burn centre group (*P* ≤ 0.007).

**Conclusion:**

Both groups differed in patient, burn, and treatment characteristics. At 12 months, quality of life and scar quality were good in both groups. Significantly poorer scar quality scores were found in the burn centre group. This might be related to their larger burns and more frequent surgery. The organization of burn care in the Netherlands seems to work adequately. Patients are treated locally when possible and are transferred when necessary.

**Supplementary Information:**

The online version contains supplementary material available at 10.1007/s00068-023-02233-9.

## Introduction

Depending on their severity, burn injuries may require treatment at a specialized burn centre. In the Netherlands, three burn centres have been designated to provide specialized care for the severely burned patients. The Emergency Management of Severe Burns (EMSB) referral criteria have been implemented since 2009, and serve as a support for non-burn centre physicians to determine which patients should be transferred to a burn centre [1].

To gain more insight into the epidemiology, injury characteristics, treatment, and outcome of burn patients, the three Dutch burn centres have established the Dutch Burn Repository (DBR R3) [2]. This repository registers all patients admitted to one of these burn centres. However, patients treated at a non-burn centre are not included in this repository, and also no other database is available to provide data regarding this specific population. Most studies focus on patients treated at burn centres rather than those treated at a non-burn centre.

Currently, there is no scientific literature available regarding the epidemiology, burn characteristics, treatment, and outcome of burn patients treated at Dutch non-burn centre hospitals. More insight in these data is crucial to improve burn care. This study was conducted to provide such data. The main aim of this study was to compare the burn injury characteristics of patients admitted to a hospital without a burn centre with patients with < 10% total body surface area (TBSA) burned who were admitted (or secondarily transferred) to the burn centre. Secondary aims were to determine whether the admissions were in agreement with the EMSB referral criteria, and to compare treatment, treatment costs, quality of life, and scar quality in these patients, until 1 year after trauma.

## Methods

### Study design and setting

This was a multicentre, prospective, cohort study. Potential participants were selected from all hospitals located in two large trauma regions in the Netherlands: the Southwest Netherlands trauma region and Network Emergency Care Brabant. The Burn Centre of the Maasstad Hospital serves as the referral centre for both region and is included in the South–West Netherlands trauma area. Of the 20 hospitals in these regions, 16 participated—2 level 1 trauma centres, 1 specialized burn centre, and 13 general hospitals. Four hospitals could not participate due to practical concerns. Patients were included between 17 September 2017 and 17 September 2019.

### Participants

Patients with burns or inhalation injury (no age limit), admitted to a hospital without a specialized burn centre in the trauma regions Southwest Netherlands or Brabant (i.e., non-burn centre group), and patients primarily admitted or secondarily transferred to the burn centre of the Maasstad Hospital with < 10% TBSA burned (i.e., burn centre group), were eligible for inclusion. Secondarily transferred patients were transferred to a burn centre within 48 h after admission, and were included in the burn centre group where they received their treatment.

A threshold of 10% was chosen to include a similar population at both types of hospitals. According to the EMSB referral criteria, none of the patients admitted to a non-burn centre hospital were expected to have burns ≥ 10% TBSA [1]. Approximately 80% of all patients admitted to a burn centre have burns < 10% TBSA [3].

Patients were excluded if (1) they died < 24 h after the trauma due to non-burn injuries (e.g., severe head injury), (2) their contact information was unknown or incomplete, or (3) they had insufficient comprehension of the Dutch or English language to understand the study documents.

Follow-up was performed at 3 weeks, and at 3, 6, and 12 months after trauma. An optional follow-up moment was performed after 6 weeks; in case, the wound had not healed (> 95% wound closure) after 3 weeks. At each follow-up visit, data were extracted from patient’s medical records and physical examination was performed. Data extraction and physical examination were performed by a trained investigator (DTVY). In addition, patients or proxies were asked to complete questionnaires at each study visit.

### Outcome measures and data collection

The primary outcome measure was the burn characteristics. Data on mechanism (e.g., scald or flame), burn injury severity (percentage TBSA burned and depth), trauma location (e.g., home or work), time to wound healing, and the presence of inhalation trauma were collected from patient’s medical records at the first study visit. The burn size and wound depth were re-evaluated by a trained investigator (DTVY) during physical examination as soon as possible after admission.

Secondary outcome measures were the adherence to the EMSB referral criteria, treatment details, treatment costs, health-related quality of life, and scar quality. Furthermore, patient characteristics and complication details (hematoma, post-operative blood loss, wound infection, pneumonia, graft loss, and edema) were collected. Additional injuries (Abbreviated Injury Score and general Injury Severity Score (ISS)) were extracted from the Dutch National Trauma Registry (NTR).

For patients primarily presented to a non-burn centre hospital or patients secondarily transferred and treated at the burn centre, the adherence to the EMSB referral criteria was determined. Non-transferred patients who met none of the referral criteria and transferred patients who met at least one of these criteria were considered as ‘appropriately transferred’. Non-transferred patients who met the referral criteria were considered as ‘undertransferred’ and transferred patients who met none of them were considered as ‘overtransferred’. The EMSB referral criteria are presented in Supplemental Table S1.

For the treatment details, data were registered regarding prehospital transportation, intubation, bronchoscopy, escharotomy, wound treatment (wound dressing or topical therapy), the type of surgery performed (e.g., split skin grafting (SSG) or full thickness skin grafting), intensive care unit (ICU) admission and length of stay, and hospital length of stay.

Health-related quality of life was measured with the EuroQol-5D-3L (EQ-5D) questionnaire. This tool consists of five items (mobility, self-care, usual activities, pain/discomfort, and anxiety/depression) and a visual analog scale (EQ-5D-VAS) for self-related health status [4]. The five separate items could be scored as: no problems, some problems, or extreme problems. The EQ-5D-VAS score ranges from 0 to 100 and a higher score indicates a better health status. The questionnaire was completed by all patients aged 4 years or older. Parents of paediatric patients aged 4–7 years old completed the ‘youth proxy’ version, and pediatric patients between 8 and 15 years old completed the ‘youth’ version by themselves or with help of their parents. This questionnaire was completed at 3 weeks (representing the pre-trauma score), and at 3-, 6-, and 12-months follow-up. For adults, a utility score was calculated, based on the five separate domains, which range from 0 to 1 in which a higher score indicates a better health status.

Scar quality was determined with the Patient and Observer Scar Assessment Scale (POSAS) in all patients. This tool consists of a patient-reported part, completed by the patient or parents of pediatric patients below 16 years of age, and an observer part, completed by a trained observer (in this study DTVY) [5]. Both parts consist of seven items (six domains and one overall score), scored from 1 (no difference with normal skin) to 10 (very different from normal skin). The mean POSAS score per patient was calculated by summing up the six items scored and dividing this by six. To gain insight into the six domains separately (see Supplemental Figure S1), the POSAS outcomes were also divided into three categories: (1) low score, no differences with normal skin: POSAS item score 1; (2) intermediate scores, minor differences with normal skin: POSAS item score 2 or 3; (3) high scores, major differences with normal skin: POSAS item score ≥ 4. These cut-off points are arbitrary in the absence of commonly used cut-off points and in the absence of a minimal important change analysis of the POSAS [6]. The POSAS was completed at 3-, 6-, and 12-months follow-up, for all wounds separately and if necessary, by or with help from parents.

All direct health care costs due to treatment, complications, and events during follow-up (e.g., surgery, admission, and follow-up visits) were determined. Costs were calculated by multiplying the volumes of health care with the corresponding unit price (Supplemental Table S2). Details on health care use were collected from patient’s medical records. Unit prices were derived from the Dutch costs manual and from two previous cost studies in burn patients [7–9]. All unit prices were adjusted for the year 2019, which was the last year of patient inclusion, using the National Consumer Price Index. Costs were reported in Euros (€1.00 equals $1.12). Mean total healthcare costs were calculated for the entire population and for the two subgroups; (1) patients treated at a non-burn centre, and (2) patients treated at the burn centre.

### Statistical analysis

Data were analyzed using the Statistical Package for the Social Sciences (SPSS) version 25.0 (SPSS, Chicago, IL, USA). Data were reported following the ‘Strengthening the Reporting of Observational studies in Epidemiology’ (STROBE) guidelines. Normality of continuous data was tested with the Shapiro–Wilk test. A *p* value < 0.05 was taken as a threshold for statistical significance in all statistical tests and all tests were two sided. Missing values were not replaced by imputation.

For continuous data (all had a non-normal distribution), median and quartiles were reported. The Shapiro–Wilk test showed that all continuous data deviated from a normal distribution. For categorical data, number and frequencies were reported. Data are shown for two separate groups: patients admitted to a non-burn centre and the patients admitted to the burn centre. Statistical comparison between groups was done with a Mann–Whitney *U* test for continuous data and with a Chi-square test for categorical data. The costs were reported as a mean with 95% confidence interval (95% CI).

## Results

### Patient selection

**Fig. 1:**
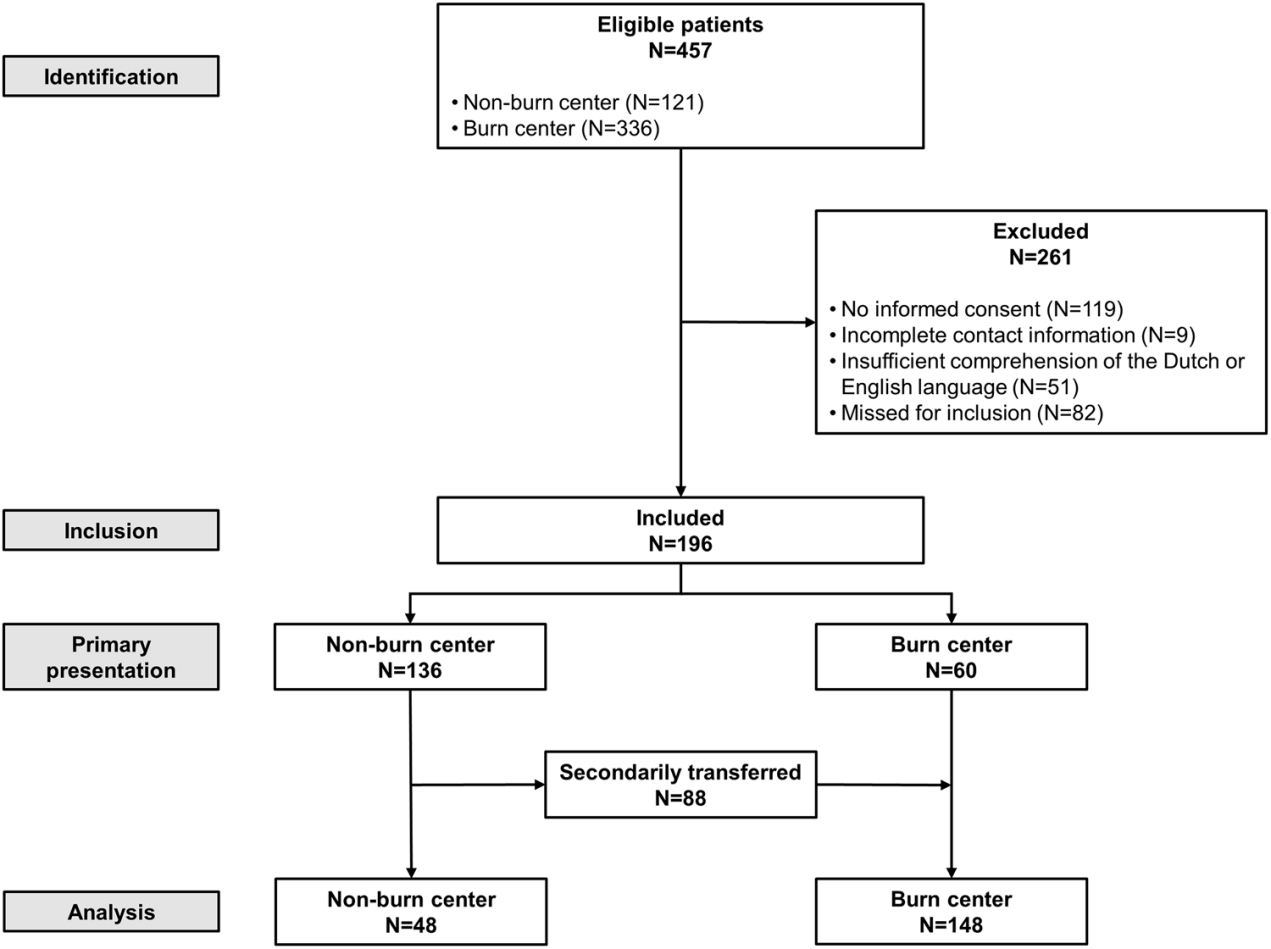
Eligibility chart

During the inclusion period, 457 patients with burns and/or inhalation injury were admitted to one of the participating hospitals. For analysis, 261 patients were excluded, leaving 196 patients included in the analysis (Fig. 1). A total of 48 patients were admitted to a non-burn centre hospital and 148 patients were primarily admitted or secondarily transferred to the burn centre. Three patients deceased shortly after admission to a non-burn centre (*N* = 2) or the burn centre (*N* = 1) due to severe inhalation injury. Fourteen patients, two from a non-burn centre and 12 from the burn centre, withdrew consent during follow-up.

### Patient characteristics and EMSB adherence

The median age in the non-burn centre group was 44 (P_25_–P_75_ 26–61) years, which was significantly higher than in the burn centre group, 30 (P_25_–P_75_ 8–52) years (*P* = 0.007; Table 1). No statistically significant differences were found between both groups regarding gender, ASA score, and comorbidity.

**Table 1:** Patient characteristics

	*N*^a^	Total (*N* = 196)	*N*^a^	Non-burn centre (*N* = 48)	*N*^a^	Burn centre (*N* = 148)	*P* value
Age (years)	196	32 (15–53)	48	44 (26–61)	148	30 (8–52)	0.007
< 1 year	196	7 (4%)	48	1 (2%)	148	6 (4%)	0.069
1–4 years	196	32 (16%)	48	4 (8%)	148	28 (19%)	
5–15 years	196	12 (6%)	48	1 (2%)	148	11 (7%)	
16–74 years	196	135 (69%)	48	37 (77%)	148	98 (66%)	
≥ 75 years	196	10 (5%)	48	5 (10%)	148	5 (3%)	
Males	196	148 (76%)	48	38 (79%)	148	110 (74%)	0.566
ASA score							
1	193	153 (79%)	45	34 (76%)	148	119 (80%)	0.805
≥ 2	193	40 (21%)	45	11 (24%)	148	29 (20%)	
Comorbidity							
Endocrine	194	13 (7%)	46	3 (7%)	148	10 (7%)	1.000
Nervous	194	10 (5%)	46	0 (0%)	148	5 (7%)	0.121
Circulatory	194	20 (10%)	46	5 (11%)	148	15 (10%)	1.000
Respiratory	194	5 (3%)	46	3 (7%)	148	2 (1%)	0.088
Mental	194	4 (2%)	46	1 (2%)	148	3 (2%)	1.000

Data are shown as median (P_25_–P_75_) or as *N* (%)

*ASA* American Society of Anaesthesiologists

^a^This represents the number of patients from whom data were available

Of the 48 patients admitted to a non-burn centre, 19 (40%) were admitted in line with the referral criteria. The remaining 29 (60%) patients met at least one of the criteria, and were considered to be undertransferred. Sixteen (55%) of the twenty-nine patients were undertransferred after consultation of the burn centre. The undertransferred patients met the following criteria: ≥ 10% TBSA burned in adults (*N* = 2), ≥ 5% full thickness burns (*N* = 2), extremes of age (*N* = 3), additional trauma or comorbidity (*N* = 4), inhalation injury (*N* = 6), functional areas (*N* = 24), chemical burns (*N* = 1), or circumferential burns (*N* = 3).

Of the 148 patients admitted to the burn centre, 60 (40%) were directly presented to the burn centre and 88 (60%) were secondarily transferred from a non-burn centre to the burn centre (within 48 h). In 68 (77%) of these 88 patients, the transferal was in line with the EMSB criteria. The remaining 20 (23%) did not meet any of the referral criteria and were considered as overtransferred. Seventeen of them (85%) were transferred after consulting a burn centre prior to referral.

### Injury characteristics

The injury characteristics per patient are presented in Table 2. The median percentage TBSA burned was significantly higher in the burn centre group [4% (P_25_–P_75_ 2–6) versus 2% (P_25_–P_75_ 1–4); *P* = 0.001]. The majority of patients (*N* = 177; 90%) had isolated burns and in both groups, scald, flame, and flash burns accounted for the greatest proportion of injuries. Injury Severity Score was significantly higher in the non-burn centre group [2 (P_25_–P_75_ 1–4) versus 1 (P_25_–P_75_ 1–1); *P* < 0.001].

**Table 2:** Injury characteristics (per patient)

	*N*^a^	Total (*N* = 196)	*N*^a^	Non-burn centre (*N* = 48)	*N*^a^	Burn centre (*N* = 148)	*P* value
TBSA burned (%)	191	4 (2–6)	45	2 (1–4)	146	4 (1–6)	0.001
Type of injury							
Burn wound	196	177 (90%)	48	39 (81%)	148	138 (93%)	0.038
Inhalation injury	196	5 (3%)	48	3 (6%)	148	2 (1%)	
Combined injury	196	14 (13%)	48	6 (13%)	148	8 (5%)	
Bronchoscopy performed	196	15 (8%)	48	6 (13%)	148	9 (6%)	0.207
Burn mechanism							
Scald	194	74 (38%)	47	14 (30%)	147	60 (41%)	0.506
Flame	194	48 (25%)	47	14 (30%)	147	24 (23%)	
Flash	194	47 (24%)	47	12 (25%)	147	35 (24%)	
Steam	194	5 (3%)	47	1 (2%)	147	4 (3%)	
Electrical	194	1 (1%)	47	0 (0%)	147	1 (1%)	
Contact	194	8 (4%)	47	4 (9%)	147	4 (3%)	
Injury Severity Score	196	1 (1–2)	48	2 (1–4)	148	1 (1–1)	< 0.001

Data are shown as median (P_25_–P_75_) or as *N* (%)

^a^This represents the number of patients from whom data were available

The enrolled patients had a total of 352 wounds; 83 (2 on average) in the non-burn centre group and 269 (2 on average) the burn centre group (Table 3). No statistically significant difference was found in wound depth on admission or in time to wound closure.

**Table 3:** Injury characteristics (per wound)

	*N*^a^	Total (*N* = 352)	*N*^a^	Non-burn centre (*N* = 83)	*N*^a^	Burn centre (*N* = 269)	*P* value
Wound location							
Head/neck	352	59 (17%)	83	11 (13%)	269	48 (18%)	0.518
Trunk	352	67 (19%)	83	17 (21%)	269	50 (19%)	
Upper extremity	352	136 (39%)	83	31 (37%)	269	105 (39%)	
Buttocks	352	5 (1%)	83	2 (2%)	269	3 (1%)	
Genitalia	352	3 (1%)	83	0 (0%)	269	3 (1%)	
Lower extremity	352	82 (23%)	83	22 (27%)	269	60 (22%)	
Depth							
Partial thickness	352	271 (77%)	83	62 (75%)	269	209 (78%)	0.681
Full thickness	352	32 (9%)	83	9 (11%)	269	23 (9%)	
Mixed (partial and full thickness)	352	49 (14%)	83	12 (15%)	269	37 (14%)	
Time to wound healing (days)	343	19 (14–32)	73	18 (13–22)	269	20 (14–32)	0.423

Data are shown as median (P_25_–P_75_) or as *N* (%)

^a^This represents the number of patients for whom data were available

### Wound treatment and hospital admission

Significantly more patients in the burn centre group underwent surgery (54 (37%) versus 7 (15%) patients; *P* = 0.004, Table 4). Split skin grafting was the most commonly performed procedure in both groups. Median hospital length of stay was significantly higher in non-burn centre patients [6 (P_25_–P_75_ 3–13) days] than in burn centre patients [2 (P_25_–P_75_ 2–5) days; *P* = 0.001]. Ten of the 48 patients from the non-burn centre group were transferred to the outpatient clinic of the burn centre for subsequent wound care after discharge.

**Table 4:** Hospital admission and treatment

	*N*^a^	Total (*N* = 196)	*N*^a^	Non-burn centre (*N* = 48)	*N*^a^	Burn centre (*N* = 148)	*P* value
ICU admission	196	26 (13%)	48	6 (13%)	148	20 (14%)	1.000
Length of stay (days)	26	2 (1–2)	6	2 (2–3)	20	2 (1–3)	0.571
Surgery performed	196	61 (31%)	48	7 (15%)	148	54 (37%)	0.004
Wound debridement	61	7 (11%)	7	5 (71%)	54	2 (4%)	0.001
Escharotomy	61	1 (2%)	7	0 (0%)	54	1 (2%)	1.000
Split skin graft	61	58 (95%)	7	5 (71%)	54	53 (98%)	0.032
Full thickness graft	61	2 (3%)	7	0 (0%)	54	2 (4%)	1.000
Wound care	61	1 (2%)	7	0 (0%)	54	1 (2%)	1.000
Staple removal	61	2 (3%)	7	0 (0%)	54	2 (4%)	1.000
Hospital length of stay (days)	196	2 (1–5)	48	6 (3–13)	148	2 (2–5)	0.001

Data are shown as median (P_25_–P_75_) or as *N* (%)

*ICU* intensive care unit

^a^This represents the number of patients from whom data were available

### Adverse events

In 24 out of 196 patients, an adverse event occurred (Table 5), with no differences between the 2 groups. Most common adverse events were wound infection and graft loss. All but one patient required additional treatment, particularly with antibiotics or surgical treatment with a split skin graft.

**Table 5:** Adverse events

	*N*^a^	Total (*N* = 196)	*N*^a^	Non-burn centre (*N* = 48)	*N*^a^	Burn centre (*N* = 148)	*P* value
Adverse event	196	24 (12%)	48	6 (13%)	148	18 (12%)	1.000
Wound infection	24	13 (54%)	6	4 (67%)	18	9 (50%)	0.879
Pneumonia	24	1 (4%)	6	0 (0%)	18	1 (6%)	
Graft loss	24	8 (33%)	6	2 (33%)	18	4 (33%)	
Edema	24	1 (4%)	6	0 (0%)	18	1 (6%)	
Hemorrhage	24	1 (4%)	6	0 (0%)	18	1 (6%)	
Treatment needed	24	23 (96%)	6	6 (100%)	18	17 (95%)	1.000
Wound dressing	23	2 (9%)	6	2 (33%)	17	0 (0%)	0.019
Surgical hemorrhage control	23	1 (4%)	6	0 (0%)	17	1 (6%)	
Antibiotics	23	13 (57%)	6	3 (50%)	17	10 (59%)	
Split skin graft	23	7 (30%)	6	1 (17%)	17	6 (35%)	
Need for hospital admission	24	8 (41%)	6	0 (0%)	18	8 (44%)	0.091

Data are shown as *N* (%)

^a^This represents the number of patients from whom data were available

### Direct health care costs

The mean total costs for patients with burn injuries were significantly higher for patients admitted to the burn centre €13 466 (95% 11,680–15,252) than for those admitted to a non-burn centre €5765 (95% 3493–8037; *P* < 0.001; Table 6). Hospital and ICU admission days accounted for the greatest proportion of costs in both groups, 69% and 76% for patients from a non-burn centre and the burn centre, respectively.

**Table 6:** Mean costs per patient

Cost category	Total	Non-burn centre	Burn centre	*P* value
Pre-hospital care	1126 (849–1402)	1096 (523–1668)	1136 (816–1455)	0.737
Admission and treatment	9811 (8379–11 243)	4459 (2254–6663)	11,547 (9873–13,221)	< 0.001
Emergency department visits	298 (285–312)	268 (248–288)	309 (293–325)	0.009
Diagnostic procedures	95 (77–113)	48 (11–85)	110 (90–131)	< 0.001
Clinical consultations	111 (87–136)	16 (0–32)	142 (112–173)	< 0.001
Surgery	607 (460–754)	137 (− 13 to 941)	760 (578–941)	< 0.001
Admission days	8699 (7341–10,057)	3990 (1906–6075)	10,226 (8624–11,827)	< 0.001
Outpatient clinic visits	643 (554–732)	210 (81–340)	783 (683–884)	< 0.001
Total	11,580 (10,057–13,104)	5765 (3493–8037)	13,466 (11,680–15,252)	< 0.001

Data are shown as mean costs per patient with 95% confidence intervals between brackets

### Health-related quality of life

**Fig. 2:**
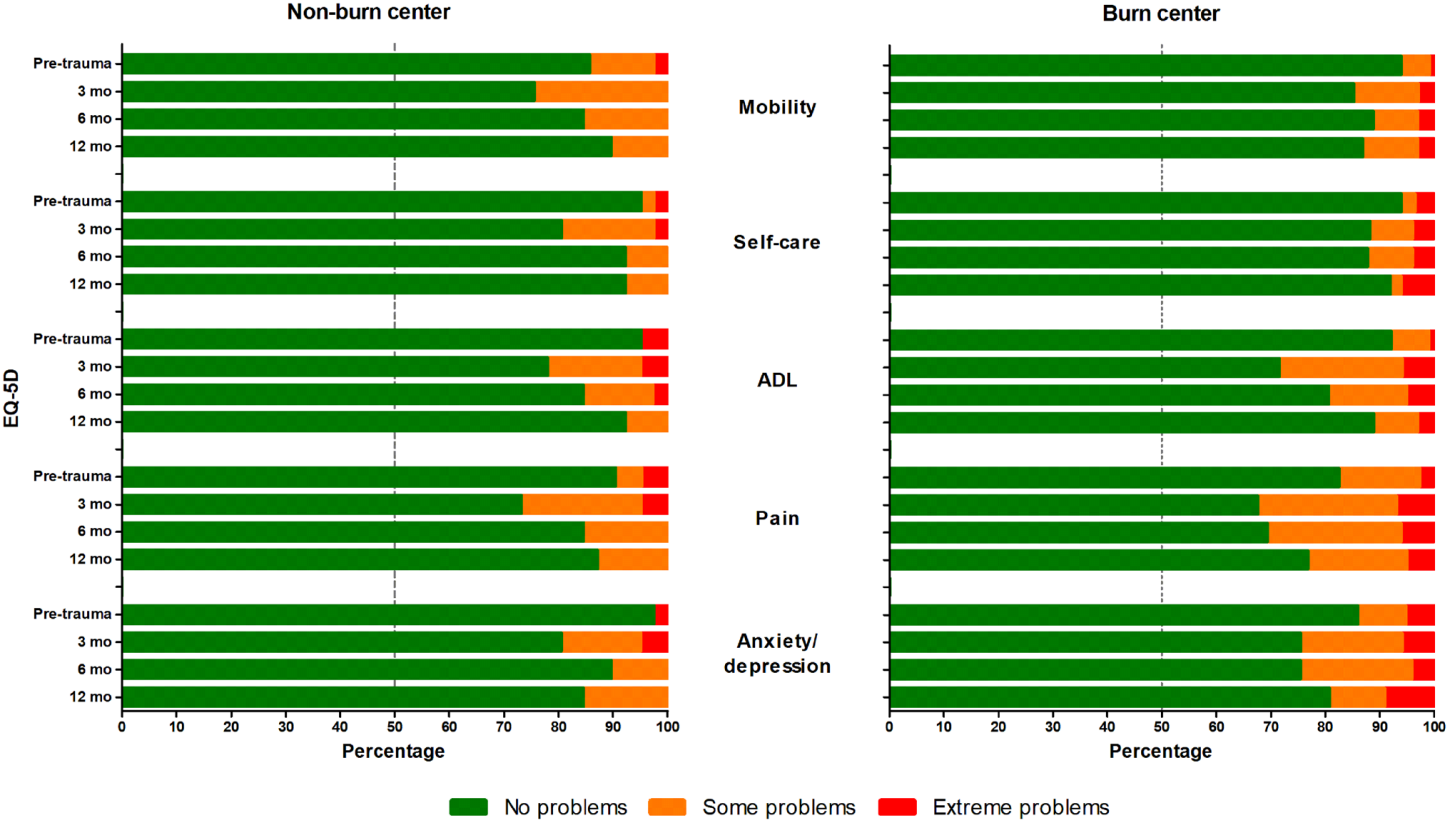
Health-related quality of life. Health-related quality of life scores, measured with the EQ-5D questionnaire, for patients from a non-burn centre and from a burn centre, at 3-, 6-, and 12-months follow-up. The vertical line at 50% represents the median score. *ADL* activities of daily living

Figure 2 shows that at 12 months, > 85% of the non-burn centre group and > 75% of the burn centre group reported no problems in any of the five health-related quality of life domains. There was no statistically significant difference between both groups. Health-related quality of life restored to pre-trauma levels in all domains, except for ‘anxiety/depression’ in non-burn centre patients, where 98% had no problems pre-trauma versus 85% at 12 months. None of the patients from the non-burn centre group experienced extreme problems at 12 months in any of the domains, compared with 5% of the patients from the burn centre group, in particular in the domains ‘anxiety/depression’ and ‘self-care’. More details are presented in Supplemental Table S3.

### Scar quality

At 12 months, the median patient and observer POSAS scores per patient (i.e., mean of the six domains combined) was < 1.5 in both groups (Table 7). Although the differences between the non-burn centre and burn centre group in POSAS patient and observer scores were statistically significant, the absolute differences were small, 0.3 and 0.1, respectively. With a median score of 2.0 (1.0–4.0), the patient and observer overall opinion scores were equal for both groups. Nevertheless, the scores were significantly higher in patients from the burn centre, reflecting a poorer scar quality. Additional details for the separate POSAS domains are shown in Supplemental Figure S1.

**Table 7:** POSAS patient and observer scores at all follow-up moments

POSAS	*N*^a^	Total (*N* = 352)	*N*^a^	Non-burn centre (*N* = 83)	*N*^a^	Burn centre (*N* = 269)	*P* value
Patient scale							
Median score							
3 months	307	2.5 (1.3–4.4)	71	1.7 (1.2–3.2)	236	2.8 (1.5–4.5)	< 0.001
6 months	307	1.8 (1.2–3.7)	70	1.3 (1.1–2.4)	237	2.0 (1.3–4.0)	< 0.001
12 months	308	1.3 (1.2–2.7)	70	1.2 (1.0–2.0)	238	1.5 (1.2–3.0)	0.007
Overall opinion							
3 months	307	3.0 (1.0–5.0)	71	2.0 (1.0–3.0)	236	3.0 (2.0–6.0)	0.002
6 months	307	3.0 (1.0–5.0)	70	2.0 (1.0–3.0)	237	3.0 (1.0–5.0)	0.004
12 months	308	2.0 (1.0–4.0)	70	2.0 (1.0–2.3)	238	2.0 (1.0–4.0)	0.037
Observer scale							
Median score							
3 months	307	1.8 (1.2–2.8)	71	1.3 (1.2–2.3)	236	2.0 (1.3–3.0)	< 0.001
6 months	307	1.5 (1.2–2.5)	70	1.2 (1.0–1.8)	237	1.7 (1.2–2.8)	< 0.001
12 months	308	1.3 (1.2–2.2)	70	1.2 (1.0–1.7)	238	1.3 (1.2–2.3)	< 0.001
Overall opinion							
3 months	307	3.0 (2.0–4.0)	71	2.0 (1.0–3.0)	236	3.0 (2.0–4.0)	0.003
6 months	307	2.0 (2.0–4.0)	70	2.0 (1.0–3.0)	237	3.0 (2.0–4.0)	0.001
12 months	308	2.0 (2.0–3.0)	70	2.0 (1.0–3.0)	238	2.0 (2.0–3.0)	0.006

Data are shown as median (P_25_–P_75_)

*POSAS* Patient and Observer Scar Assessment Scale

^a^This represents the number of wounds from whom data were available

## Discussion

This study found that patients from the non-burn centre group were older, had higher ISS, and had smaller burns. Patients from the burn centre group underwent more surgeries and were admitted shorter, but had higher health care costs involved. At 12-months follow-up, the majority of all patients reported no problems in health-related quality of life, which was equal in both groups. The median POSAS score was low in both groups, reflecting good scar quality. However, it was significantly higher in burn centre patients.

Although certain inclusion criteria were chosen in this study to include two homogeneous cohorts, significant differences were found in parameters that may affect the outcomes, such as a higher TBSA burned and more surgeries performed in the burn-centre group. Several sub-analyses were performed to find an explanation for the higher surgery rate, but no clues were found. It is possible that more severely injured patients were treated at the burn centre. This was reflected by a higher TBSA in this group, but not in wound depth. It is commonly known that it is difficult to assess wound depth by eye, even for trained physicians. For this reason, the Laser Doppler Imaging is good alternative and most often used instrument in burn centres to objectify wound depth [10]. Maybe, there was an actual difference in wound depth present, but it was not possible to objectify this with the instruments used in this study. Therefore, wound depth is a potential confounder to the data. Another very well possible explanation for the higher surgery rate in the burn centre is that burn centre physicians are more used to and comfortable with surgical treatment of burns, and therefore is more commonly done. Nevertheless, it is possible that differences in patient/injury/treatment characteristics affect the outcome parameters; therefore, one should be careful in drawing firm conclusions when comparing both groups.

It was an interesting finding that the duration of hospital admission was significantly shorter in the burn centre group, while the health care costs were much higher. Possibly, burn centre physicians are more comfortable in treating patients at the outpatient clinic, and also surgeries are often performed in a day care setting. They can wait at home for their surgery and are mostly discharged the same day. Although the admission days were lower in the burn centre group, the total costs were higher. The greatest proportion of the burn centre cost are the admission days, which are about twice as high compared with a general hospital, resulting in a higher total cost for the burn centre group (see Supplemental Table S2). Admission to a burn centre is relatively expensive, because burn care takes a lot of personnel and time per patient, it also provides for extra personnel and paramedics, and also covers for the facilities that have to be available 24/7.

Due to the improvement of burn care, more and more patients survive their burn injuries [11, 12]. This has shifted the focus of burn care from survival to quality of life. As a result, health-related quality of life and scar quality score have become important parameters for treatment and research purposes [13–15]. This study shows that outcomes were good in both groups for quality of life and scar quality. More than 80% reported to have no problems in any of the five health domains and the median scar quality scores were nearly equal to normal skin (all scores were ≤ 1.5). Since this is the first study to present data from patients treated at a non-burn centre, it is not possible to compare our non-burn centre results with other literature. Regarding the burn centre group, health-related quality of life found in our study is comparable with a meta-analysis, which also reports that approximately 80% of burn patients (median 7% TBSA) reported no problems in any of the EQ-5D domains > 12 months after trauma [16]. Also scar quality was good in comparison to other studies. In a comparable cohort of children and adults with mild-to-intermediate burns, Spronk et al. reported a median POSAS parent score of 2.7 and a median POSAS patient score of 3.0 [6, 17]. In a population with minor burns, the mean POSAS patient score was 2.4 and the observer score was 1.9, 18 months after trauma [18].

The POSAS questionnaire is one of the best available instruments to measure scar quality that has been used widely in burn research [19]. Commonly used cut-off points, or a ‘minimal important change’ for the POSAS score, have not been determined yet. In our study, differences in scar quality were small, varying from 0.1 to 1.0 on the POSAS. A recent study by Legemate et al*.* (unpublished data) found that the minimal important change of the POSAS had poor discriminative value and poor correlation with clinical change. Therefore, it remains unclear whether the difference found in this study also reflects a clinically meaningful difference.

An interesting finding was that 60% of the patients in the non-burn centre group met at least one of the EMSB referral criteria, and therefore were considered as undertransferred. This is comparable with our previous study conducted between 2014 and 2018, where 50% of the patients who received final treatment at a non-burn centre were undertransferred [20]. In daily practice, there can be various reasons to deviate from the guidelines. In 55% of the undertransferred patients, a burn centre physician was consulted for treatment advice. When these patients are not taken into consideration, the number of undertransferred patients would decrease to 27%. This is exemplary of how the referral criteria should be applied, and enables local physicians to treat patients locally whenever possible, and to transfer to a burn centre when necessary. Furthermore, two patients in this current study were not transferred because they were moribund on admission and nine were advised to visit the outpatient clinic of the burn centre for subsequent treatment. The question rises whether not following the referral criteria harms patient outcomes. Although this was not a research question, sub-analysis showed that scar quality at 12 months was good in patients who were not transferred while they met one of the EMSB criteria (mean POSAS patient and observer were 1.3 and 1.2, respectively; data not shown in text). Although the number of patients in these analyses was low, these analyses suggest that not strictly following the referral criteria did not harm the study patients. This, in combination with the number of patients that are discussed with a burn centre physician, shows that the triage and referral system in the Netherlands seems to work adequately, and that it is permissible to deviate from the guideline in specific cases.

To optimize the referral criteria and gain a higher adherence rate, more research should be done to determine which of the specific criterion should be followed more or less strictly. Also, adjusting some of the criteria would be an improvement. For example, burn location should be related to burn severity, such as depth and size. This way only patients who sustained burns that might give complications during treatment or after treatment (contractures) are transferred to a burn centre. In case of doubt, patients should be discussed with a burn physician through telemedicine. Creating such a network would be a step in creating a more patient-tailored treatment plan.

### Strengths and limitations

This is the first prospective study that provides insight into the epidemiology and patient-reported outcomes of patients treated at a non-burn centre hospital. By including patients from 15 non-burn centre hospitals and 1 burn centre, an as complete as possible overview of the distribution, treatment, costs, and outcome of burn patients admitted in two trauma regions was given.

A limitation of this study is the relatively low number of patients in the non-burn centre group, which also limits the abilities of stratified analysis of, among others, age and injury location. Unfortunately, 36 patients out of 121 from the non-burn centre group were missed for inclusion. Furthermore, although the inclusion and exclusion criteria were tailored to include two homogenous groups, some selection bias may have occurred resulting in two heterogeneous groups which hampers proper comparison. Also bias due to inclusion of undertransferred and overtransferred patients in the analysis cannot be ruled out. However, the number of patients in the correctly managed groups was too low for subgroup analysis.

## Conclusion

This study was conducted because no data are available in current literature on the outcome of patients treated in a non-burn centre hospital. This study found that patients admitted to a non-burn centre were older, had a higher overall Injury Severity Score, and had smaller burns than patients admitted or secondarily transferred to the burn centre. No differences could be observed in burn depth, yet significantly more surgeries were performed in patients from the burn centre. Also, the treatment costs were significantly higher in the burn centre group. After 1 year, outcomes in terms of health-related quality of life and scar quality were good in both groups.

## Supplementary Information

Below is the link to the electronic supplementary material.

Supplementary file1 (DOCX 142 KB)

Supplementary file2 (TIF 4487 KB)

## Declarations

### Availability of data and material

Anonymized data analyzed for the current study will be shared if a reasonable request is made by a qualified investigator to the corresponding author.
