# Supplementary material for: Burn injury characteristics, referral pattern, treatment (costs), and outcome in burn patients admitted to a hospital with or without a specialized Burn Centre (BURN-Pro)
Source: Eur J Trauma Emerg Surg. 2023 Feb 3;49(3):1505–15. doi: 10.1007/s00068-023-02233-9 (PMC10229686; doi:10.1007/s00068-023-02233-9)
Supplement: Supplementary file 1 — Supplementary file1 (DOCX 142 KB) [file 68_2023_2233_MOESM1_ESM.docx]

# SUPPLEMENTAL FILES

## Supplemental Figure S1. POSAS patient and observer scores

This figure shows the scar quality outcome score, measured with the POSAS, for patients from a non-burn centre (A&C) and from the burn centre (B&D), at 3-, 6-, and 12-months follow-up. The vertical line at 50% represents the median score.

POSAS, Patient and Observer Scar Assessment Scale.

## Supplemental Table S1. Emergency Management of Severe Burns referral criteria; adjusted for Dutch hospitals

| Burns 10% or more TBSA in adults |
| --- |
| Burns 5% or more TBSA in children (<16 year) |
| Full Thickness burns 5% or more TBSA |
| Burns of functional areas – face, hands, feet, genitals, perineum, or large joints (*i.e.*, shoulder, elbow, knee, and ankle) |
| Circumferential burns of the neck, chest, or extremities |
| Electrical burns (high voltage) including lightning strikes |
| Chemical burns |
| Burns with suspected associated inhalation injury |
| Any burn patient with associated trauma or (pre-existing) medical condition that may affect treatment and recovery, or could increase mortality |
| Burns at the extremes of age – young children (<1 year) and the elderly (≥75 years) |
| Non-accidental burns |
| Burns for which the burn mechanism is uncertain in combination with uncertainty about the competence/equipment of the hospital for these types of injuries |
| Burn wound that show insufficient signs of healing within two weeks |

TBSA, Total Body Surface Area.

## Supplemental Table S2. Sources and unit costs of health care resources

| **Cost category** | **Unit** | **Source of**  **consumption data** | **Source of**  **unit prices (cost year)** | **Unit price (in €, 2019)** |
| --- | --- | --- | --- | --- |
| **Pre-hospital costs** |  |  |  |  |
| Own transportation | Ride | Study registry | Hop *et al.* 2016 (2014) [9] | Upon request |
| Ambulance | Ride | Study registry | NZa (2018)^a^ | 702.00 |
| HEMS-assistance | Yes | Study registry | Hospital data (2008) | Upon request |
| **Admission and primary treatment** |  |  |  |  |
| Emergency department | Visit | Hospital registry | Cost manual (2014)^b^ | 259.00 |
| Diagnostic procedures |  |  |  |  |
| Laser Doppler Imaging | Scan | Study registry | Hop *et al.* 2016 (2012) [8] | 151.00 |
| Bronchoscopy | Procedure | Study registry | Hop *et al.* 2016 (2003) [8] | 301.00 |
| Clinical consultations |  |  |  |  |
| Medical photographer | Visit | Hospital registry | Hop *et al.* 2016 (2012) [8] | 40.35 |
| Physical therapist | Visit | Hospital registry | Hop *et al.* 2016 (2012) [8] | 15.33 |
| Social worker | Visit | Hospital registry | Hop *et al.* 2016 (2012) [8] | 33.73 |
| Occupational therapist | Visit | Hospital registry | Hop *et al.* 2016 (2012) [8] | 12.26 |
| Medical specialist | Visit | Hospital registry | Hop *et al.* 2016 (2012) [8] | 35.97 |
| Surgery |  |  |  |  |
| Pre-operative consultation | Visit | Hospital registry | Hop *et al.* 2016 (2009) [8] | 52.00^1^ |
| Operation room | Hours | Hospital registry | Hop *et al.* 2016 (2012)^c^ [9] | 708.02^1^/441.30^3,d^ |
| Surgeon | Hours | Hospital registry | Cost manual (2014)^b^ | 81.00 |
| Admission days |  |  |  |  |
| Intensive Care Unit | Days | Hospital registry | Cost manual(2014)^b^/Hop *et al.* 2016 (2012) [9] | 1186.00^1,2^/2966.00^3^ |
| General hospital | Days | Hospital registry | Cost manual (2014)^b^ | 443.00 |
| Academic hospital | Days | Hospital registry | Cost manual (2014)^b^ | 642.00 |
| Burn centre | Days | Hospital registry | Hop *et al.* 2016 (2012) [8] | 948.00 |
| Day care burn centre | Day | Hospital registry | Hop *et al.* 2016 (2012) [9] | 349.60 |
| **Outpatient clinic visits** |  |  |  |  |
| Wound care | Visit | Hospital registry | Cost manual (2010)^b^/Hop *et al.* 2016 (2012) [8] | 28.36^1,2^/60.79^3^ |
| Medical photographer | Visit | Hospital registry | Hop *et al.* 2016 (2012) [8] | 40.35 |
| Scar care | Visit | Hospital registry | Hop *et al.* 2016 (2012) [8] | 67.96 |
| Medical specialist | Visit | Hospital registry | Cost manual (2010)^b^ | 80.00^1,3^/163.00^2^ |
| Plastic surgeon burn centre | Visit | Hospital registry | Hop *et al.* 2016 (2012) [9] | 71.94 |
| Physical therapist | Visit | Hospital registry | Hop *et al.* 2016 (2012) [8] | 43.54 |
| Skin therapist | Visit | Hospital registry | Hop *et al.* 2016 (2012) [8] | 39.27 |
| Psychologist | Visit | Hospital registry | Hop *et al.* 2016 (2012) [8] | 61.59 |
| After care nurse | Visit | Hospital registry | Hop *et al.* 2016 (2012) [8] | 39.27 |

Unit prices presented in the last column of this table are adjusted to 2019 by using the national consumer price index.

HEMS, Helicopter Emergency Medical Service.

^a^ NZa, Nederlandse Zorgautoriteit [in English: Dutch Healthcare Authority): Standard costs prices, online available on: https://puc.overheid.nl/nza/doc/PUC_254194_22/.

^b^ Costing manual: Methodology of costing research and reference prices for economic evaluations in healthcare, version 2016 [7].

^c^ Excluding 41.90% overhead costs.

^d^ Per surgical procedure €674.55 is added as fixed costs for pre-operative consultation, nursing personnel, and instruments.

^1^ General hospital.

^2^ Academic hospital.

^3^ Burn centre.

## Supplemental Table S3. Health-related quality of life at all follow-up moments

|  |  |  | **Total**  **(N=196)** |  | **Non-burn centre**  **(N=48)** |  | **Burn centre**  **(N=148)** | **P-value** |
| --- | --- | --- | --- | --- | --- | --- | --- | --- |
|  |  | **N^*^** |  | **N^*^** |  | **N^*^** |  |  |
| **EQ-5D-3L score (adults)** |  |  |  |  |  |  |  |  |
| Utility score | Pre-trauma | 140 | 1.0 (0.9-1.0) | 40 | 1.0 (1.0-1.0) | 100 | 1.0 (0.8-1.0) | 0.155 |
|  | 3 months | 128 | 1.0 (0.8-1.0) | 39 | 1 (0.8-1.0) | 89 | 1.0 (0.7-1.0) | 0.598 |
|  | 6 months | 122 | 1.0 (0.8-1.0) | 37 | 1.0 (0.9-1.0) | 85 | 1.0 (0.7-1.0) | 0.065 |
|  | 12 months | 124 | 1.0 (0.8-1.0) | 37 | 1.0 (0.9-1.0) | 87 | 1.0 (0.8-1.0) | 0.152 |
| Visual Analog Scale | Pre-trauma | 140 | 85 (80-90) | 40 | 84 (80-90) | 100 | 85 (75-90) | 0.953 |
|  | 3 months | 128 | 80 (70-85) | 39 | 80 (75-88) | 89 | 80 (65-80) | 0.108 |
|  | 6 months | 122 | 80 (70-90) | 37 | 80 (73-90) | 85 | 80 (70-89) | 0.084 |
|  | 12 months | 124 | 80 (70-90) | 37 | 80 (75-90) | 87 | 80 (70-90) | 0.495 |
| **EQ-5D-3L score (children)** |  |  |  |  |  |  |  |  |
| Mobility | Pre-trauma | 16 | 0 (0%) | 2 | 0 (0%) | 14 | 0 (0%) | N/A. |
|  | 3 months | 15 | 1 (7%) | 2 | 0 (0%) | 13 | 1 (8%) | 1.000 |
|  | 6 months | 15 | 0 (0%) | 2 | 0 (0%) | 13 | 0 (0%) | N/A |
|  | 12 months | 14 | 0 (0%) | 2 | 0 (0%) | 12 | 0 (0%) | N/A |
| Self-care | Pre-trauma | 16 | 0 (0%) | 2 | 0 (0%) | 14 | 0 (0%) | N/A |
|  | 3 months | 15 | 0 (0%) | 2 | 0 (0%) | 13 | 0 (0%) | N/A |
|  | 6 months | 15 | 1 (7%) | 2 | 0 (0%) | 13 | 1 (8%) | 1.000 |
|  | 12 months | 14 | 0 (0%) | 2 | 0 (0%) | 12 | 0 (0%) | N/A |
| Usual activities | Pre-trauma | 16 | 0 (0%) | 2 | 0 (0%) | 14 | 0 (0%) | N/A |
|  | 3 months | 15 | 2 (13%) | 2 | 0 (0%) | 13 | 2 (15%) | 0.743 |
|  | 6 months | 15 | 0 (0%) | 2 | 0 (0%) | 13 | 0 (0%) | N/A |
|  | 12 months | 14 | 0 (0%) | 2 | 0 (0%) | 12 | 0 (0%) | N/A |
| Pain/discomfort | Pre-trauma | 16 | 0 (0%) | 2 | 0 (0%) | 14 | 0 (0%) | N/A |
|  | 3 months | 15 | 2 (13%) | 2 | 0 (0%) | 13 | 2 (15%) | 1.000 |
|  | 6 months | 15 | 1 (7%) | 2 | 0 (0%) | 13 | 1 (8%) | 1.000 |
|  | 12 months | 14 | 0 (0%) | 2 | 0 (0%) | 12 | 0 (0%) | N/A |
| Anxiety/depression | Pre-trauma | 16 | 0 (0%) | 2 | 0 (0%) | 14 | 0 (0%) | N/A |
|  | 3 months | 15 | 2 (13%) | 2 | 0 (0%) | 13 | 2 (15%) | 1.000 |
|  | 6 months | 15 | 2 (13%) | 2 | 0 (0%) | 13 | 2 (15%) | 1.000 |
|  | 12 months | 14 | 1 (7%) | 2 | 0 (0%) | 12 | 1 (8%) | 1.000 |
| Visual Analog Scale | Pre-trauma | 16 | 90 (86-100) | 2 | 100 (100-100) | 14 | 90 (84-99) | 0.112 |
|  | 3 months | 15 | 90 (85-100) | 2 | 98 (95-100) | 13 | 85 (83-95) | 0.140 |
|  | 6 months | 15 | 90 (80-100) | 2 | 93 (90-100) | 13 | 85 (80-100) | 0.603 |
|  | 12 months | 14 | 93 (84-100) | 2 | 95 (90-100) | 12 | 90 (82-99) | 0.400 |

Data are shown as median (P_25_-P_75_) or as N (%).

**^*^** This represents the number of patients from whom data were available.

N/A, Not applicable
