# Supplementary figures and images for: Burn injury characteristics, referral pattern, treatment (costs), and outcome in burn patients admitted to a hospital with or without a specialized Burn Centre (BURN-Pro)
Source: Eur J Trauma Emerg Surg. 2023 Feb 3;49(3):1505–15. doi: 10.1007/s00068-023-02233-9 (PMC10229686; doi:10.1007/s00068-023-02233-9)

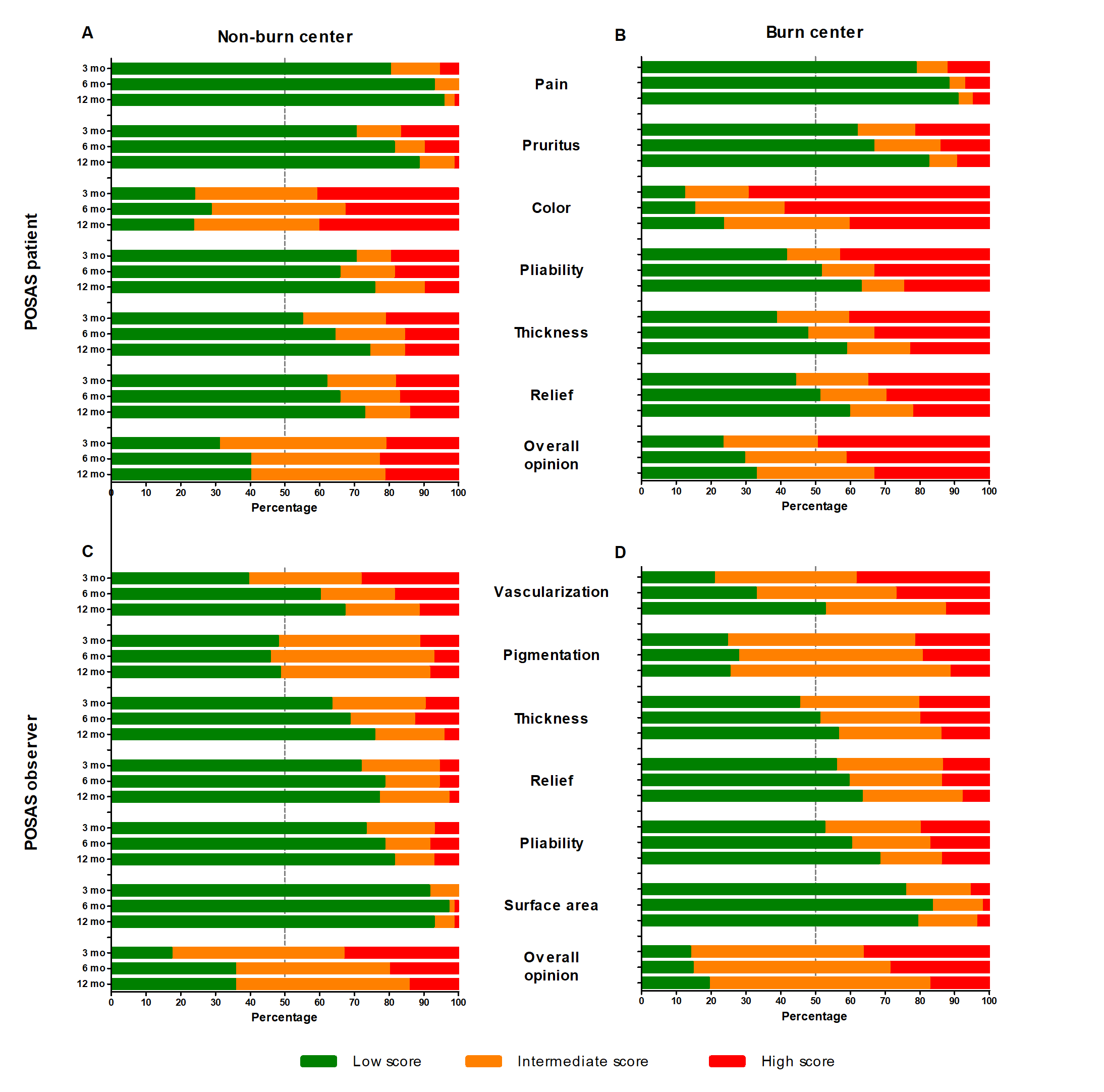

Supplement: Supplementary file 2 — Supplementary file2 (TIF 4487 KB) [file 68_2023_2233_MOESM2_ESM.tif]
